# Development of an autonomously replicating viral expression system tailored for *Catharanthus roseus*

**DOI:** 10.1111/pbi.13274

**Published:** 2019-11-12

**Authors:** Cara Mortimer, Benjamin Dugdale, Peter Waterhouse

**Affiliations:** ^1^ Centre for Tropical Crops and Biocommodities Queensland University of Technology Brisbane 4000 Queensland Australia

**Keywords:** viral vector, *Catharanthus roseus*, gene expression, MIA, viblastine, vincristine, monoterpinoid indole alkaloid, transient gene expression

*Catharanthus roseus* is an important medicinal plant with a capacity to synthesize >130 monoterpinoid indole alkaloids (MIA). Many of these compounds, including the chemotherapeutics vinblastine, vincristine and their chemical derivatives have high economic value with diverse applications. Vincristine is the only effective antileukaemic drug that can drastically reduce white blood cell counts. Since the 1950s, it has increased the survival rate of children with leukaemia from 20% to 80% (Nejat *et al.*, 2015). However, due to its scarcity, vincristine is one of the most expensive plant‐derived compounds on the market and supply cannot meet demand (Kumar *et al.*, 2013). Total chemical synthesis of vinblastine and vincristine is inefficient due to their structural complexity and stereochemistry and industrial production relies on the extraction of precursors from *C. roseus* in which the MIAs accumulate in trace amounts; for example 5.0 and 0.5 ppm for vinblastine and vincristine, respectively. Accordingly, recent attention has shifted to the development of new approaches to elevate MIA levels in *C. roseus*. The MIA biosynthetic pathway, however, is intricate and composed of around 30 to 50 enzymatic steps with multifaceted metabolic regulation involving intracellular compartmentalization, different cell types and tissue differentiation, making it a complex target for metabolic engineering (Dugé de Bernonville *et al*., 2015, Nejat *et al.*, 2015). Furthermore, unravelling the MIA biosynthetic pathway and its regulation in *C. roseus* has been hindered by a paucity of technologies to express candidate genes *in planta*. With the exception of a virus‐induced gene silencing (VIGS) system (Liscombe and O'Connor, 2011), genomic approaches that enable gene functions to be explored *in vivo* have been most effective in (and often limited to) hairy root and cell suspension cultures. These systems lack the required level of cyto‐ and tissue‐differentiation essential for the expression of MIA pathway genes and, therefore, do not truly reflect MIA biosynthesis and its regulation *in planta* (Liscombe and O'Connor, 2011). To overcome this, we have utilized the replication machinery of a *C. roseus‐*infecting geminivirus to develop an autonomously replicating viral expression system that provides consistent and high gene expression in *C. roseus* leaves*.*

Geminiviruses are small, circular, single‐stranded DNA viruses that infect monocotyledonous and dicotyledonous plants in tropical and subtropical regions. The family *Geminiviridae* is divided into nine genera, of which the *Begomovirus* genus is the largest consisting of both monopartite and bipartite genome types (Hanley‐Bowdoin *et al.*, 2013). Geminiviruses replicate their genomes exclusively in the nucleus of the infected host cell via a rolling circle replication process (Figure 1b) initiated at a consensus stem loop structure located in the intergenic region (IR). The IR also serves to direct divergent transcription of both virion and complementary sense genes. A coat protein (CP) forms the viral capsid and mediates vector transmission, V2 and AV2 function as anti‐defence proteins to inhibit post‐transcriptional gene silencing (PTGS) and the Replication initiator protein (Rep) initiates viral replication. Begomoviruses encode three additional gene products, the transcriptional activator protein (TrAP; and the related C2 protein) interfere with transcriptional gene silencing (TGS) and PTGS, the replication enhancer protein (REn) is involved in viral replication and the C4 protein counteracts PTGS (Hanley‐Bowdoin *et al.*, 2013). In 2012, the complete nucleotide sequence of a bipartite begomovirus was isolated from *C. roseus,* the virus was named Catharanthus yellow mosaic virus (CaYMV) (Ilyas *et al.*, 2013).

**Figure 1:**
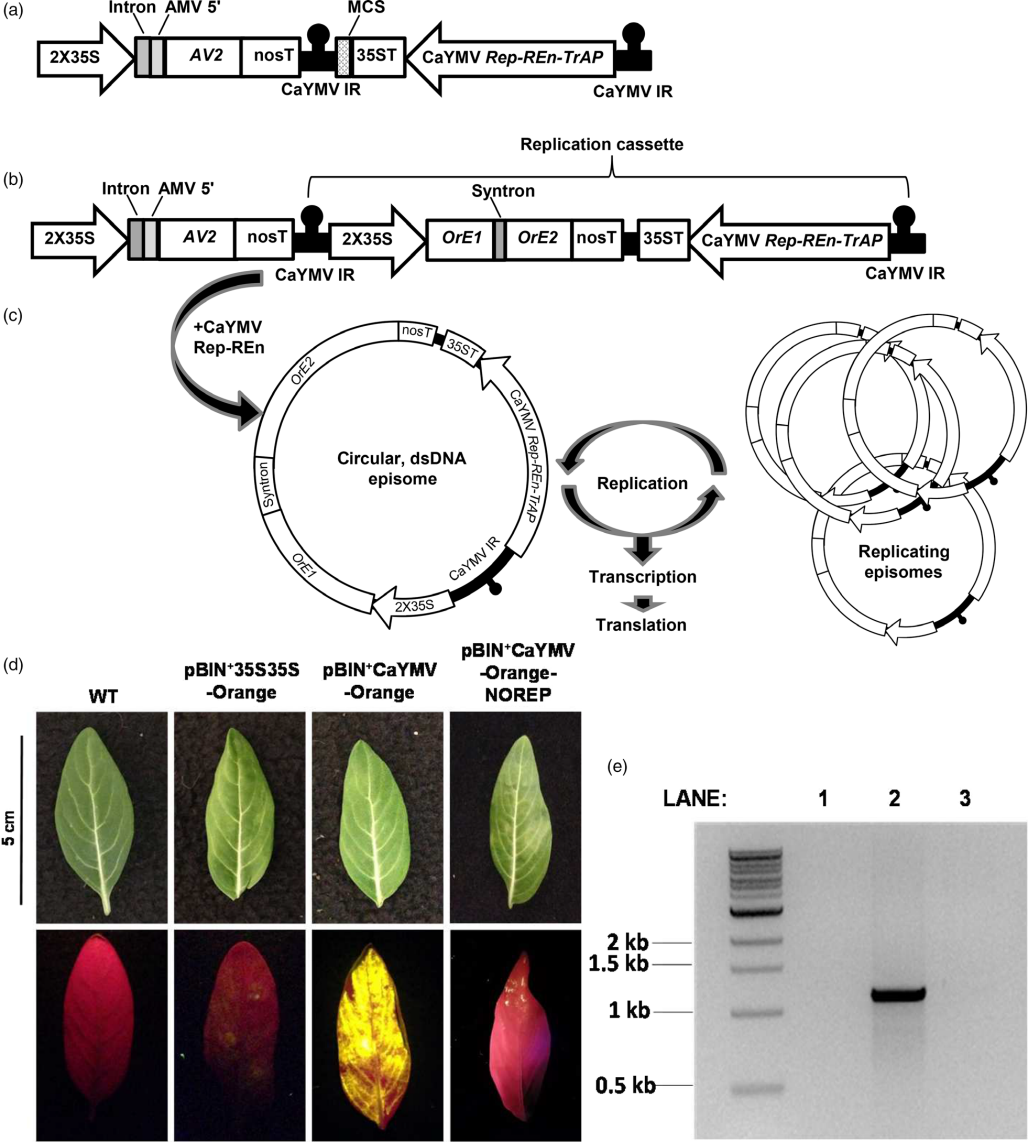
Development of an autonomously replicating viral expression system tailored for *Catharanthus roseus* **.** An autonomously replicating viral expression system for *C. roseus* was constructed and its efficacy demonstrated using the fluorescent reporter protein Orange. (a) The Catharanthus yellow mosaic virus (CaYMV)‐based system was mobilized into binary plasmid pBIN^+^creating a ~4 kb vector pBIN^+^CaYMV. (b) An *Orange* expression cassette was inserted into the MCS creating a ~5.5 kb vector pBIN^+^CaYMV‐Orange. (c) The region between the IR repeats (the replication cassette ~4 kb including *Orange*) serves as a template for rolling circle replication initiated by CaYMV Rep. This results in a circular, single‐stranded DNA episome, which is converted to a double‐stranded (ds) form by host polymerases. The dsDNA episomes are transcriptionally active and serve as a template for further amplification. (d) pBIN^+^CaYMV‐Orange was agroinfiltrated into *C. roseus* and expression visualized 6 days after infiltration. Fluorescence was compared to (i) wild‐type (WT) leaves, (ii) leaves infiltrated with a non‐replicating vector (pBIN^+^35S35S‐ORANGE) and (iii) leaves infiltrated with a CaYMV vector in which the Rep gene was disrupted (pBIN^+^CaYMV‐Orange‐NOREP). (e) DNA was extracted from infiltrated leaf tissues and probed for replication by PCR: Lane 1, WT DNA; Lane 2, pBIN^+^CaYMV‐Orange; Lane 3, pBIN^+^CaYMV‐Orange‐NOREP. Intron, potato *gbbs* gene intron IV; 2X35S, enhanced CaMV 35S promoter; *AV2*, CaYMV anti‐defence protein coding sequence; nosT and nopaline synthase terminator; AMV 5′, UTR translational enhancer sequence from alfalfa mosaic virus; MCS, multiple cloning site; 35ST CaMV 35S terminator; CaYMV *Rep‐Ren‐TrAP*, CaYMV replicase, replication enhancer and transcriptional activator coding regions; *OrE1/OrE2* Exons 1/2 of *Orange*.

We designed a CaYMV‐based replicating vector system for transient gene expression *in C. roseus*; illustrated in Figure 1a. The system contains the CaYMV *Rep*, *Ren* and *TrAP* genes under the transcriptional control of their native promoter sequences and located between repeats of the CaYMV IR. A cauliflower mosaic virus (CaMV) 35S 3′ UTR was inserted downstream of *REn* to effectively terminate transcription. The *CP* gene was not included in order to render the system non‐infectious. Outside the replicative unit, but within the T‐DNA, was located an expression cassette containing the CaYMV *AV2* gene under the transcriptional control of an enhanced CaMV 35S promoter (2X35S), potato *gbbs* gene intron IV and alfalfa mosaic virus translational enhancer. The *AV2* gene was included in the expression system based on its anti‐defence role during virus infection. *AV2* was placed outside of the replication unit to moderate its expression as AV2 proteins can trigger a hypersensitive response (Mubin *et al.*, 2010). A multiple cloning site (MCS) was included in the replication unit in order to insert the transgene of interest.

The CaYMV‐based cassette was chemically synthesized and mobilized into the binary plasmid pBIN^+^ to generate vector pBIN^+^CaYMV (Figure 1a). An expression cassette containing an *Orange* florescent protein reporter gene sequence and synthetic intron (syntron) under the transcriptional control of a 2X35S promoter was inserted into pBIN^+^CaYMV as an *Asi*SI‐*Avr*II restriction fragment to create vector pBIN^+^CaYMV‐Orange (Figure 1b). The syntron was included to prevent Orange expression in Agrobacteria. Following Rep‐initiated replicative release, the single‐stranded, circular episomal molecule is converted to a double‐stranded DNA form via the origin of second strand synthesis and by host polymerases (Figure 1c). This form is both transcriptionally active and serves as a template for further rolling circle amplification resulting in high episomal copy number and high transgene expression levels. The vector pBIN^+^CaYMV‐Orange was mobilized into *Agrobacterium tumefaciens* (strain AGL1) by electroporation and agroinfiltrated into *C. roseus* leaves via the lower epidermis with a needle‐less syringe, at an OD_600_ of 1.5*.*

Transient expression of Orange in *C. roseus* leaves agroinfiltrated with pBIN^+^CaYMV‐Orange was assessed by visualizing florescence with a Dual Florescent Protein (DFP) flashlight and a Royal Blue LED and Yellow filter. Six days after agroinfiltration, fluorescence was compared to wild‐type (WT) tissue and leaves infiltrated with a non‐replicating vector (pBIN^+^2X35S‐ORANGE). Expression of Orange was significantly higher and more uniform in *C. roseus* leaves agroinfiltrated with pBIN^+^CaYMV‐Orange (Figure 1d). To prove that high level Orange expression was the result of Rep‐mediated replication of the expression unit, the *Rep* gene sequence in pBIN^+^CaYMV‐Orange was disrupted and the *REn* coding sequence removed, creating vector pBIN^+^CaYMV‐Orange‐NOREP. Minimal Orange florescence was observed in leaf tissue agroinfiltrated with this vector (Figure 1d). To demonstrate replicative release from the vector and the production of CaYMV‐Orange episomal DNA forms, outwardly extending primers were designed to amplify a 1100 bp sequence spanning the CaYMV IR. These primers were used in a PCR with DNA extracted from leaves agroinfiltrated with pBIN^+^CaYMV‐Orange and pBIN^+^CaYMV‐Orange‐NOREP, 6 days after agroinfiltration (Figure 1e). No PCR product was obtained from WT leaves (Figure 1e, Lane 1). A strong PCR product of ~1100 bp was amplified from leaves agroinfiltrated with pBIN^+^CaYMV‐Orange suggesting extra‐chromosomal episome formation and Rep‐mediated amplification, whereas a very weak ~1100 bp product was amplified from leaves agroinfiltrated with pBIN^+^CaYMV‐Orange‐NOREP, indicating host‐mediated recombination and low‐level episome formation, independent of Rep (Figure 1e, Lanes 2 and 3, respectively).

We have designed an autonomously replicating viral expression platform based on the genome of CaYMV and tailored for high and consistent gene expression in the leaves of *C. roseus.* Up to this point, the lack of a reliable transgene expression system in this host has been a limitation to better understanding and manipulating MIA biosynthesis and regulation. The CaYMV system could, for example, be used to express candidate genes involved in the transport of MIA intermediates between plant cells and organs, such as a recently proposed but as yet uncharacterized secologanin transporter (Kidd *et al.*, 2019). The platform described here opens up new pathways in MIA research and product development. Our approach also highlights how viruses can be exploited to develop tailored gene expression systems for specific species that are recalcitrant to established gene expression technologies.

## Competing interests

The authors have no competing interests.

## Contributions

C.M., B.D. and P.W. designed the experiments, C.M. performed the experiments and analysed the results. CM and BD wrote the manuscript. All authors read and approved the final manuscript.
